# Challenges of conflict of interest, coordination and collaboration in small island contexts: towards effective tobacco control governance in UK Overseas Territories

**DOI:** 10.1136/tobaccocontrol-2021-057077

**Published:** 2022-01-25

**Authors:** Rachel Ann Barry, Sarah E Hill, Sarah Williams, Jeff Collin

**Affiliations:** 1 School of Social and Political Science, University of Edinburgh, Edinburgh, UK; 2 Sydney School of Public Health, The University of Sydney, Sydney, New South Wales, Australia; 3 Department of Health and Social Care, Office for Health Improvement and Disparities (formerly Public Health England), London, UK; 4 Global Health Policy Unit, The University of Edinburgh School of Social and Political Science, Edinburgh, UK

**Keywords:** tobacco industry, public policy, global health, globalisation

## Abstract

**Introduction:**

The UK Overseas Territories (UKOTs) are semi-autonomous jurisdictions that face distinctive challenges in implementing tobacco control and protecting policy from industry influence. They are not eligible to become independent parties of the WHO Framework Convention on Tobacco Control (FCTC), although they can apply for treaty extension under the UK’s ratification. This study explores the relevance of the FCTC—particularly Article 5.3—for tobacco control governance across a sample of UKOTs.

**Methods:**

From March to May 2019, we interviewed 32 stakeholders across four territories (Anguilla, Bermuda, Cayman Islands, St Helena) at diverse stages in implementing key FCTC measures. Thematic qualitative analysis explored awareness and perceptions in relation to tobacco control.

**Results:**

Interviewees’ accounts highlight the complexity of protecting health policy from industry influence in a context where the ‘tobacco industry’ covers a diverse range of actors. Despite not being formally covered by the FCTC, several health officials spoke about the strategic value of invoking Article 5.3 in the context of tensions with economic priorities. Nevertheless, effective tobacco control governance is complicated by territories’ reliance on local businesses—including tourism—and close social connections that occasionally blur the lines between private and public spheres.

**Conclusions:**

The UKOTs share many characteristics with other small island jurisdictions, creating distinctive challenges for advancing tobacco control and protecting policy from industry interference. Despite their complex status in relation to WHO and its architecture, these territories benefit from the norms embedded in the FCTC and the systems that support its implementation.

## Introduction

The United Kingdom’s Overseas Territories (UKOTs) comprise 14 jurisdictions^1^ (Anguilla; Bermuda; British Antarctic Territory; British Indian Ocean Territory; British Virgin Islands; Cayman Islands; Falkland Islands; Gibraltar; Montserrat; Pitcairn, Henderson, Ducie and Oeno Islands; St Helena, Ascension and Tristan da Cunha; South Georgia and South Sandwich Islands; Turks and Caicos Islands; and Sovereign Base Areas of Akrotiri and Dhekelia in Cyprus) that are largely self-governing but neither independent states nor members of WHO.^2^ While represented by the UK in international contexts (including UN organisations), the territories are not automatically covered by treaties and legal instruments ratified by the UK. The majority of territories are not formally covered by the WHO Framework Convention on Tobacco Control (FCTC), although ratification was extended to Gibraltar in 2020^3^ and there is interest among other territories in obtaining similar extension.^4^ Formal coverage requires territories ‘to demonstrate that the necessary domestic provisions are in place to support extension’.^5 6^

Existing tobacco control measures are mixed, with some territories not yet having any specific regulations and others recently introducing quite comprehensive legislation.^4^ The UK government has worked to support territories in strengthening their tobacco control policies via its Conflict, Stability and Security Fund, which aims to promote good governance and sustainable development^7^ and ‘to ensure the UK’s international health obligations are met’.^8^ This support has focused on strengthening implementation of Articles 8, 11 and 13 of the FCTC by advancing legislation to deliver smoke-free public places, appropriate labelling of tobacco products and prohibition of tobacco advertising respectively.^6^

With a combined population around quarter of a million,^5^ the UKOTs’ issues in acceding to the FCTC may seem of marginal interest to global health. Yet the UKOTs share key social, economic and political attributes with around one-fifth of the countries that have ratified the FCTC, namely small island developing states (SIDS)^9^; while indirect engagement with the FCTC is also shared by other non-member jurisdictions such as Hong Kong,^10^ Macao,^11^ the Cook Islands and Niue.^3^ Most of the 38 states categorised as SIDS by the UN^12^ have ratified the FCTC (exceptions are Cuba, Haiti and the Dominican Republic), while several Pacific islands are associate members lacking full UN status. SIDS and small non-state jurisdictions share governance challenges relevant to tobacco control, including geographical isolation, small and highly connected societies, a narrow economic base, distinctive political and cultural sensitivities and limited governmental capacity and human resources.^13–19^ A small literature on Pacific Island countries further illustrates the significance of ‘islandness’ to effective tobacco control,^20 21^ including in managing conflicts of interest and preventing tobacco industry interference.^22–24^

The UKOTs provide an opportunity to examine the *de facto* strategic value of the FCTC in strengthening tobacco control governance prior to incurring *de jure* obligations under formal ratification. We focus here on the most pressing priority for advancing FCTC implementation^25^—namely minimising tobacco industry interference (Article 5.3^26^)—alongside commitments to coordinated multisectoral approaches (Article 5.1 and 5.2) and international collaboration (5.4 and 5.5). The UKOTs’ lack of independent statehood poses challenges for the latter, although the Pan-American Health Organization (PAHO) is committed to raising awareness of the FCTC and its provisions^27^ among the Caribbean territories (Anguilla, Bermuda, British Virgin Islands, Cayman Islands, Montserrat, Turks and Caicos Islands).

This study aims to explore awareness of the FCTC and its relevance for effective tobacco control across a sample of UKOTs, with particular focus on Article 5.3. Drawing on interviews from four UKOTs, we examine understandings of the tobacco industry in remote island contexts and consider related challenges to developing coordinated multisectoral strategies. We explore the significance of the FCTC and international collaborations for those working to advance tobacco control amid geographical isolation, limited public sector capacity and complex social and political systems.

## Methods

As part of a wider study examining tobacco control in the UKOTs,^4^ interviews were conducted with stakeholders relevant to tobacco control efforts across four territories, that is, Anguilla, Bermuda, Cayman Islands and St Helena. These territories were selected on the basis that all four governments had signalled interest in strengthening tobacco control, yet the territories were at different stages in implementing key FCTC measures. While tobacco industry interference potentially impacts all areas of tobacco control, the wider study focused on FCTC Articles 8, 11 and 13 as areas in which there was a particular need for policy development in these contexts.^6^ In these respects, Bermuda and Cayman Islands had the most developed tobacco control frameworks during the interview process, including comprehensive bans on smoking in indoor public places, restrictions or bans on tobacco advertising and requiring health warnings on tobacco packaging; St Helena had partial measures in place on smoke-free environments and advertising bans, but no requirements for health warnings on tobacco packaging and Anguilla had no specific tobacco control legislation (table 1).^4^

**Table 1:** Implementation of key tobacco control measures (FCTC Articles 8, 11 and 13) in study territories at the time of interviews

Territory	Population (year estimated)	Smoking prevalence in adults (year estimated)	Tobacco control legislation	Implementation of key FCTC articles		
				Article 8 (smoke-free environments)	Article 11 (health warnings/packaging)	Article 13 (ban on advertising)
Anguilla	16 300 (2010)	5.8% (2018)	None	–	–	–
Bermuda	64 700 (2011)	13.9% (2014)	Tobacco Control Act 2015	Comprehensive	Text warning (30%)	Heavily restricted
Cayman Islands	55 500 (2010)	15% (2012)	Tobacco Control Law 2008 Tobacco Regulations 2010	Comprehensive	Text warning (30%)	Complete prohibition
St Helena	5000 (2010–2)	22.2% (2021)	Tobacco Control Ordinance 2011	Partial	No	Via UK law*

Smoking prevalence: Anguilla Ministry of Health and Social Development, 2018^43^; Government of Bermuda, 2016^44^; Cayman Islands Government, 2012^45^; St Helena, 2021^46^; Tobacco control legislation: Bermuda Tobacco Control Act, 2015^47^; Cayman Islands Tobacco Law, 2008^48^; Cayman Islands Tobacco Regulations, 2010^49^; English Law (Application) Ordinance 2005^50^; St Helena Tobacco Control Ordinance, 2011.^51^

Sources: Population estimates: UK Foreign & Commonwealth Office, 2012.^52^

*Subject to UK Tobacco Advertising and Promotion Act 2002.^53^

FCTC, Framework Convention on Tobacco Control.

We sought to interview a range of relevant stakeholders—including civil servants, healthcare workers, representatives of health charities and local community leaders. Interviewees were identified using purposive sampling and approached via the civil servant with lead responsibility for tobacco control within each territory. All interviews but one were carried out with stakeholders based in the relevant UKOTs; the exception being conducted with a representative of a regional non-governmental organisation (NGO) engaged in tobacco control.

A total of 32 semi-structured interviews were carried out from March to May 2019, with half of all interviewees working as civil servants (particularly in health policy), over a quarter working in healthcare or health-related charities and one-fifth representing local community leaders (including politicians, business representatives and a teacher) (table 2). Interviews in Bermuda (12) and the Cayman Islands (6) were conducted in person, as was the interview with the regional NGO official, while those in Anguilla (6) and St Helena (7) were conducted remotely via telephone or digital audio-conferencing.

**Table 2:** Interviewees by location and role

Interviewee location	Number
Anguilla	6
Bermuda	12
Cayman	6
St Helena	7
Other	1
Total	32
**Interviewee role**	**Number**
Civil servants:	16
*Health policy*	*8*
*Health protection/health education*	*4*
*Other*	*4*
Healthcare worker	5
Health charity	4
Community leaders:	7
*Politician*	*4*
*Business*	*2*
*Teacher*	*1*
Total	32

Interviewee numbers are not disaggregated by location and role in order to maintain anonymity.

All interviews were audio-recorded and transcribed with the interviewee’s consent. Interviews varied in length from 20 to 68 min, with an average duration of approximately 45 min. We coded interview transcripts in NVivo V.12 using a thematic framework developed iteratively through repeated readings of transcripts. Coded data were then used to develop a narrative analysis, examining awareness of both the FCTC and tobacco industry activity and their relevance for efforts to advance tobacco control across the four UKOTs.

## Results

### Awareness and relevance of the FCTC

Within UKOT governments, awareness of the FCTC was limited to those civil servants directly engaged in tobacco control. Such officials showed generally strong awareness of the treaty and regarded it as a valuable benchmark for tobacco control actions. Commitment to the treaty was understood as a prerequisite for its extension to the territories, and there was a desire among health policy advisors to ‘tick as many and all of the boxes around FCTC’ and ‘try to fill the FCTC gaps’ based on ‘exactly what it is that is required’. The FCTC was presented as an external frame of reference for tobacco control legislation. As explained by one health official:

…when we were drafting the law, that was our guiding document, and so that’s why we’ve been able to draft the law in such a way that it’s compliant with almost all the articles, so that was our guiding tool, our guiding document.

Alongside embodying international best practice, the FCTC was invoked by policymakers as offering political leverage in justifying decisions affecting the business community. Interviewees who had worked on tobacco legislation suggested they could ‘almost blame the FCTC’ in responding to local business concerns by presenting compliance in terms of ‘this is what we have to do’. In the words of one health official:

It sort of helps us to be able to say, ‘this is the best practice’…[] Particularly for decision-makers when they’re being faced with potentially having to make a decision that might not be popular with some of their constituents in the business community, to have something like the FCTC to say ‘this is what the UK is committed to, this is what people around the world, governments around the world are looking at’.

### Article 5.3, ambiguous understandings of the tobacco industry and conflict of interest

The FCTC’s political functions extended to aiding management of industry interference, notwithstanding generally limited awareness of Article 5.3. One official described addressing members of the Chamber of Commerce and “tell(ing) them to their faces, ‘no, we didn’t consult you because the FCTC said I don’t have to consult you’”. Another civil servant described using Article 5.3 to limit their health ministry’s interactions with industry actors:

This is where the WHO framework came into play. The WHO framework specifies that you do not consult with industry stakeholders because they have a way of trying to navigate around and see how they can circumvent [proposed restrictions]. I think that mistake was made by the ministry once, and then when I started drafting the legislation it stopped.

Awareness of tobacco industry interference in the UKOTs was similarly restricted to those with direct experience in developing new legislation. In Bermuda and Cayman Islands (territories that had recently passed tobacco control legislation), health officials described how representatives of transnational tobacco companies had attempted to block proposed measures. One tobacco lead noted the potentially damaging impact of such interference:

We weren’t even confident [the draft legislation] was going to get put back on the agenda because we were having all this pressure from the outside, like I told you, British American Tobacco, Japan Tobacco, Philip Morris. They were in Bermuda, they were sending us lawyers’ letters… they were asking for private meetings with the minister and were advising the minister not to [support it].

In considering prospects for introducing standardised packaging of tobacco products, the same interviewee expressed concern that the tobacco industry “may tie us up in knots, if we decide to [go ahead with it], spend some real money to make an example of us. And we don’t have the money”.

In contrast, most interviewees saw tobacco transnationals as lacking interest in the UKOTs (given their small populations), and regarded their territory as free from industry influence. A handful of respondents (with experience of developing tobacco legislation) recognised diverse faces of the tobacco industry and the potential for local commercial actors to advance wider industry interests. One civil servant engaged in developing legislation saw the main sources of ‘pushback’ as being ‘retail, Chamber of Commerce especially. The owners of the smoke shops, retailers selling it, wholesalers especially’; while a health policy advisor was similarly less anxious about opposition from tobacco transnationals and ‘more concerned about the shopkeepers, even the hotels’.

In some territories, political sensitivities were heightened by specific instances of local, small-scale tobacco production. In the Cayman Islands, interviewees expressed concern about tobacco being locally grown at a residential rehabilitation centre as a way of generating revenue. Several participants in Bermuda mentioned a local retailer (the ‘Smoke Shop’) that imported loose-leaf tobacco and manufactured cheap cigarettes for local sale. A local politician expressed concern about the impact of tobacco regulations on local retailers (“the ‘ma and pa’ stores”) that sold single cigarettes in low-income neighbourhoods but were seen as supporting the local community rather than acting to protect industry interests. Similar perceptions shaped several interviewees’ understandings of appropriate interactions in policy contexts. Some health officials spoke of the desirability of involving local retailers, importers and businesses on legislative advisory committees, which were presented as spaces where policymakers could negotiate a balance between health and economic priorities. As one tobacco lead explained:

We wanted to get their input as a tobacco dealer, and how it would affect them, what we could do to create compromises. The main thing was whether or not it was going to affect the businesses.

In contrast, interviewees experienced in developing tobacco legislation saw local importers, retailers and chambers of commerce as acting on behalf of the tobacco industry—as reflected by a local politician:

I mean, there was resistance. I remember being lobbied, in fact […] being lobbied by the tobacco industry. What it really was, was one of the biggest distributors of Bermuda, importers of tobacco products, and he had us come to his office and spoke about [proposed tobacco control legislation]. I walked out thinking, I’m being lobbied by tobacco.

Such interviewees referred explicitly to conflicts of interest and the desirability of excluding industry-affiliated actors from policy discussions. However, they also recognised practical constraints arising from the territories’ economic reliance on tourism and hospitality. In discussing how to address such tensions, one health official noted, “it’s not going to be easy. I don’t know how it’s going to work because of the relationship that all the leaders have with these entities, these hotels”. Another civil servant asked “which politician is going to tell the big fancy [tobacco importer] giving him millions of dollars to run their old campaigns—that they can’t do this and they can’t do that?” Managing government-industry interactions was particularly difficult where key politicians were financially invested in the tobacco industry. In describing politicians’ reluctance to advance tobacco control, an interviewee from a local charity explained “I think there’s a couple of [politicians] that are either wholesale importers or they are retailers”.

### Challenges of promoting coordination and coherence

The challenge of limiting government-industry interactions is compounded by a perceived tension between tobacco control and territories’ economic reliance on tourism and hospitality.^16^ According to one health official, “everything that is big business is related to tourism in some shape or form […] we don’t have any other investments; it’s the only industry, the only way to make money”. Such tensions add to challenges in developing a coordinated whole-of-government approach to tobacco control. Several health officials identified the need to work across policy silos, highlighting how effective tobacco control and non-communicable disease (NCD) prevention required a coordinated approach. In the words of one tobacco lead: “Because we realise that chronic disease interventions are beyond health…we [need to] get all sectors involved, all persons involved”. Interviewees working in health policy expressed a desire for more intersectoral engagement, arguing that ‘having agencies like the Drug Council who are pushing from other directions and helping also to educate the public is really important as well’.

In practice, however, tobacco control efforts were largely confined to health with limited engagement across sectors such as commerce, education, customs and drug policy. One civil servant working in drug prevention was unaware of their territory’s tobacco policies, while another noted that tobacco control had ‘primarily been a feature of the public health department’ with a lack of joined-up policy: “the tobacco framework, while we put it into legislation, when we develop other public health policies, other drug policies, it’s not being tied in”. Several interviewees would welcome more intersectoral engagement, which was often seen as a consequence of limited public sector capacity. An interviewee from a local charity recalled a multistakeholder committee being created to advance a piece of legislation, but such coordination was rare and ad hoc, being established for a specific time-limited purpose and then dissolved. One health official described a general sense that different departments ‘do not work well together’ with a lack of ‘inner-working’ across diverse parts of government.

As indicated above, human resource constraints—characteristic of small island contexts^13 15 18^—were seen as limiting scope for intersectoral coordination. Lack of capacity was frequently cited, with one civil servant outside of health describing their department as “a staff of six or seven, expected to do all of this, but we certainly don’t have the funding to … have the capacity to inform”. Staffing issues were also seen as an obstacle to intersectoral collaboration in enforcement, as described by a health protection officer:

We are probably understaffed here, and we have other concerns. I think you also have to collaborate a lot with customs, that will be your first line of defence so to speak. And I know right now there’s not a lot of—I should say there’s not any like customs officers responsible for port health, there’s not that sort of training within that staff.

Interviewees spoke of reliance on personal connections in compensating for the lack of formal coordinating structures, with high levels of social connection seen as providing an alternative means of informal communication. As one civil servant noted:

Because we are so close knit, you know, we have weekly meetings where we will discuss it and say, oh, by the way did you know? And they will pick it back up again. They will say, oh, we will send an email to the ministry, which is totally outside of [own department]. But because we’re such a small jurisdiction, we understand, okay, well, maybe these guys forgot about it or maybe they didn’t think about [doing that].

### Significance of international cooperation to tobacco control

Alongside limited intersectoral collaboration, tobacco control officials often reported experiencing professional isolation—which could lead to feelings of being ‘dragged down’ and ‘discouraged’. Officials particularly valued opportunities for international engagement and support—opportunities that are constrained by the UKOTs’ lacking statehood and full WHO membership status. Interviewees spoke of the benefits of regional links, notably those created via PAHO and Public Health England (PHE). One tobacco lead talked of the UKOTs’ inclusion in a PAHO workshop as “the first time we were all meeting together and we learned so much from each other”. Another interviewee spoke of the encouragement colleagues derived from learning that ‘actually small islands have done it (tobacco control) and have done it successfully’, adding that—on return to their home territory—the relevant politicians ‘had this renewed zeal about’ advancing tobacco control measures.

Meetings with other UKOTs and Caribbean states provided spaces where health officials could exchange knowledge and share practices for responding to their contextual challenges. One health official discussed the importance of lesson learning in navigating complex conflicts of interest generated by close relationships between politicians and industry actors:

We have to realise we live in the Caribbean, we’re political […] those persons that finance tobacco were approaching the politicians to [say], ‘I will finance your campaign, I will give your island X amount towards education, however, you need to ease some pressure’. So because you know that was coming, even before that conversation started […] when you went to executive council […] you could have showed them the pros and the cons [of engaging with industry-funded donors], because you already had that [awareness].

Support from WHO and its regional offices was similarly seen as important for territories’ efforts to advance tobacco control. In the case of St Helena—which is more isolated than the Caribbean territories and lacks a regional ‘peer group’—policymakers benefited substantially from a WHO-funded visit to Mauritius. A local politician described the visit as enabling them “to look at the patterns of chronic diseases, what support measures they had in place, and see what we could take from them in terms of lessons learned”.

Interactions with WHO are constrained by the distinctive legal status of the UKOTs. Nevertheless, health officials in the Caribbean territories clearly regarded their ‘longstanding’ relationship with PAHO as invaluable. There was a sense that ‘PAHO is our grandfather’, providing technical and political support and linking health officials in the territories with counterparts in other Caribbean jurisdictions. Alongside PHE, cooperation with PAHO was seen as relevant in seeking extension of FCTC coverage. PAHO’s engagement with the territories was perceived as offering leverage for tobacco control across different sectors—as explained by another health official:

PAHO sends us a questionnaire annually and says, well, here’s the MPOWER, you know, how do you rate yourself? […] this questionnaire arrives and then you have to circulate [it] to colleagues and customs and they’re like, ‘Oh, this is serious, this is from PAHO’. Then, you actually have to get data. Yes, it’s great. It’s fantastic.

Alongside such regional interactions, officials emphasised the benefits of engagement with the international tobacco control community. One tobacco lead described the revelatory power of participation in the World Conference on Tobacco or Health:

[I]t was amazing to see everybody reporting on the FCTC and recognising what a powerful convention that is. Then, some of these countries that were standing up and saying, you know, ‘tobacco-free Ireland’. Some of the Scandinavian countries, Finland maybe, was just like, oh, my god. It was amazing. Yes, it was almost like a religious experience. You’re like, holy cow, this is public health.

## Discussion

Stakeholder accounts from four UKOTs highlight the challenges of advancing tobacco control in small island jurisdictions and the FCTC’s value in supporting local efforts of health officials. Our interviews illustrate the complexity of protecting health policy from industry interference in contexts where understandings of ‘the industry’ cover diverse actors and close social connections can blur the lines between private and public spheres. While lacking the status of full member states, UKOTs nevertheless benefit from engagement with both WHO and treaty frameworks as sources of political leverage and technical guidance.

The UKOTs’ experiences are salient in understanding challenges of implementing the FCTC—particularly Article 5.3—in small and resource-constrained settings. Few interviewees were aware of industry efforts to influence policy, reflecting economic contexts in which industry interests are mediated through local commercial actors while the transnational manufacturers of ‘Big Tobacco’ seem more remote. Regulating interactions with local businesses as part of the tobacco industry is likely to be politically sensitive, as reflected in Antigua’s and Barbuda’s tobacco legislation, which excludes wholesalers and distributors in defining the tobacco industry.^28^ Yet, tobacco industry interference via local actors is potentially significant, as recognised by WHO in jurisdictions such as the Marshall Islands.^29^

These data also demonstrate the FCTC’s strategic value beyond its official scope. Our interviewees provide a distinctive account of the FCTC’s significance for policy debates outside its formal jurisdiction—thus demonstrating the treaty’s wider significance in non-ratifying countries, as also indicated by the USA’s inclusion of many FCTC provisions in the Family Smoking Prevention and Control Act,^30^ Taiwan’s emphasis on its tobacco control policies being ‘constantly updated and aligned to international standards’^31^ and the FCTC’s policy relevance to health advocates in Indonesia.^32^ As an authoritative codification of international best practice, the FCTC can be invoked in efforts to advance tobacco control and as a lobbying device to marginalise opposition. This supranational dimension extends the treaty’s policy significance beyond what was captured by the WHO FCTC Impact Assessment Expert Group.^25^ Extensive ratification means evaluation naturally focuses on the now 182 parties^3 33^ that are legally bound to implement the treaty’s provisions; but a comprehensive understanding of its impact on international tobacco control would encompass the FCTC’s strategic value beyond these countries.

One particularly interesting finding is UKOT officials reportedly invoking Article 5.3 to minimise tobacco industry interference in policy discussions. Given widespread challenges of implementing Article 5.3,^25 34 35^ it is perhaps surprising that officials were able to use it in contexts not formally covered by the FCTC. While this partly reflects ambiguities regarding the UKOTs’ position within UK treaty obligations, it also underlines how Article 5.3 has promoted the norm of industry exclusion as fundamental to tobacco control.^36^ The UKOTs’ distinctive legal status arguably exacerbates the challenges intrinsic to small island states. Statehood provides opportunities for support and engagement that are circumscribed for the territories. Interactions with PAHO are particularly valued by tobacco control officials working in the Caribbean territories, while PHE funding and technical support has catalysed progress towards FCTC implementation and extension.

Our data highlight the extent to which governance challenges in the UKOTs are shaped by distinctive island contexts. Consistent with the SIDS policy literature, our findings underline how geographical isolation and a narrow economic base empower local business interests, particularly the tourism industry.^16 17 37^ This influence is reinforced by high levels of social connectivity.^15 18^ The entanglement of public and private roles^19^ is exemplified by lawmakers having interests in the tobacco industry via local businesses—as illustrated in our interviewees’ accounts of politicians’ links with local hotels, retailers and distributors via personal connections, political contributions or even ownership. At the same time, coordination in tobacco control policy—and NCD prevention more broadly—is challenged by limited human resources, precluding comprehensive whole-of-government approaches. Thus, our findings illustrate the institutional constraints inherent in small bureaucracies, where a handful of officials cover several policy areas and often find themselves ‘wearing multiple hats’ across public and private spheres.^13 14^

Understanding the challenges of managing tobacco industry interference within UKOTs can help inform whole-of-government, multisectoral approaches to tackling wider NCD burdens in SIDS.^38 39^ Such synergies are illustrated by PAHO’s work to strengthen conflict of interest management in nutrition policy^38^ and by civil society and policymakers’ efforts to tackle NCDs in the Caribbean^28^ and by the Pacific community’s commitment to improved monitoring of tobacco industry interference alongside linked challenges in alcohol and nutrition policy.^24^ The 2021 SIDS Summit for Health signalled the importance of tackling the commercial determinants of health for reducing NCD burdens,^40^ while reiterating the necessity of interventions being appropriate to island contexts and ‘adapted to challenges and constraints faced’.^41^ This study underlines the need for further research to understand how experience with Article 5.3 can inform capacity building to regulate a broad range of commercial determinants of health across SIDS, given their limited human resources and distinctive political economies.

This research is limited by its focus on four UKOTs, with fewer interviews in some compared with others. We were able to interview key health officials in each territory, however, and high levels of social connectivity (plus the enthusiastic support of local tobacco leads) provided exceptional access to other relevant stakeholders (the only individuals who declined an invitation to interview were direct employees of the UK government). Ideally, all interviews would have been conducted in person, but logistical constraints required some to be carried out remotely.

Supporting small islands is a priority for FCTC implementation, given they constitute one-fifth of all parties to the convention. Our findings add to the few existing studies highlighting the difficulties of minimising tobacco industry interference^22 23 28^ alongside the wider challenges posed by ‘islandness’ to effective tobacco control.^20 21^ Such evidence underlines the need to support officials in promoting good health governance and managing conflicts of interest in such contexts.^28^ Our findings also highlight the significance for small jurisdictions of engaging with international tobacco control—reinforcing the importance of supporting diverse participation in the FCTC Conference of Parties.^42^ Finally, sustained financial support and capacity building are needed to strengthen tobacco control governance and to accelerate action on NCD prevention in small island contexts.

**What this paper adds**

**What is already known on this subject**

- Small island jurisdictions face distinctive challenges in implementing effective tobacco control and protecting health policy from industry interference.
- As non-state jurisdictions, most UK Overseas Territories (UKOTs) do not participate as independent members in WHO and its associated instruments—including the WHO Framework Convention on Tobacco Control (FCTC).

**What this paper adds**

- In small island contexts, efforts to protect tobacco control governance from industry interference are complicated by the diverse faces of the ‘tobacco industry’ including the significance of local businesses and limited visibility of tobacco transnationals.
- As an authoritative codification of international best practice, the FCTC—including Article 5.3—has strategic value beyond jurisdictions that have formally acceded to it.
- Small island jurisdictions such as the UKOTs benefit from engagement in the international tobacco control community and from support for FCTC implementation.

## Data Availability

No data are available. Interviews were conducted in confidence and interview transcripts are not available outside the study team.
